# A Global Model for Bankruptcy Prediction

**DOI:** 10.1371/journal.pone.0166693

**Published:** 2016-11-23

**Authors:** David Alaminos, Agustín del Castillo, Manuel Ángel Fernández

**Affiliations:** Department of Finance and Accounting, Universidad de Málaga, Málaga, Spain; Universidad de Alicante, ITALY

## Abstract

The recent world financial crisis has increased the number of bankruptcies in numerous countries and has resulted in a new area of research which responds to the need to predict this phenomenon, not only at the level of individual countries, but also at a global level, offering explanations of the common characteristics shared by the affected companies. Nevertheless, few studies focus on the prediction of bankruptcies globally. In order to compensate for this lack of empirical literature, this study has used a methodological framework of logistic regression to construct predictive bankruptcy models for Asia, Europe and America, and other global models for the whole world. The objective is to construct a global model with a high capacity for predicting bankruptcy in any region of the world. The results obtained have allowed us to confirm the superiority of the global model in comparison to regional models over periods of up to three years prior to bankruptcy.

## Introduction

This study focuses on predicting the risk of the bankruptcy of businesses with an international scope. The current importance of bankruptcy prediction models has grown due to the recent world financial crisis. This crisis has seen an increase in the number of bankruptcies in several countries [1,2] and has served to demonstrate that even the best international companies have to be continuously vigilant concerning their financial situation and the position of the companies they work [3].

On the other hand, due to the globalisation process that the world economy is experiencing, a complex network of international relationships has arisen in the business world [4]. Some studies have shown that the globalisation phenomenon has brought about the homogenisation of the financial behaviour of companies, methods of finance, and the behaviour of financial markets [5–7]. This has also resulted in a new area of research, given the need to create models to predict bankruptcy, not just for a given country, but also to explain the common features shared by companies in the same geographical setting [8]. However, when creating models that attempt to offer rigorous predictions of bankruptcy, the majority of these have centred on companies in a single country or industry [9–12] or have focused on comparing the results of different predictive models, but without considering the creation of a global model [8].

Globally, the implications of the development of new bankruptcy prediction models are currently increasing. A large number of quoted companies operate in several countries, which means differences between them are reduced, regardless of their location or the factors particular to the country of origin. Nonetheless, few studies have focused on the global prediction of bankruptcy. In order to compensate for the marked lack of global models to predict bankruptcy, in this study we have used a logistical regression methodological framework, with the construction of regional models for Asia, Europe and America and further global models. From this standpoint, the aim of our research has been to verify whether global models have a high capacity to predict bankruptcy in any region of the world. [13] verified the fact that regional models are more accurate than global models when predicting future financial difficulties for companies, but did not consider bankruptcy. An approach to the concept of business insolvency from the perspective of financial difficulties or alternatively from the perspective of bankruptcy, may give very different results.

This study is organised as follows: section 2 contains a review of existing literature on bankruptcy prediction; section 3 describes the methodology used; section 4 considers the variables selected and the samples required for the creation of the models; section 5 then presents the results obtained. Finally, we present the main conclusions of the study, its limitations, and future lines for research.

## Literature Review and Research Hypotheses

The principal questions dealt with by the literature on insolvency prediction have been to determine which ratios or variables to include in the models and to evaluate which analysis technique is most effective for predictive purposes. In this regard, the research relies on advances in statistics and computational techniques, allowing the formulation of models with greater predictive power. This is perhaps the reason why insolvency prediction literature, given the absence of a global theory explaining the phenomenon of failure, has seen a marked increase parallel to the evolution of the analytical techniques used.

The first studies concentrate on so-called pure individual classifiers. These include statistical classifiers, such as the individual analysis of variables, multidiscriminant analysis (MDA) and discrete choice modelling, which are both straightforward and easy to use. The individual analysis of variables was the first method used in solvency prediction. [14] proposed two methods for the analysis of individual variables called profile analysis and the univariable discriminant model. Via profile analysis of the five-year period prior to bankruptcy, he discovered that the results for financial ratios in groups of firms were significantly different, with these differences becoming even more noticeable as the moment of bankruptcy approached.

After [14], a great deal of literature has been written in this area. [15] carried out the first important research into prediction through the selection of financial ratios, creating a model called Z-score. This model has a predictive accuracy of 95% using the MDA method. For his part, [16] used logistic regression (Logit) in order to create a bankruptcy prediction model for US companies. [17] also estimated the probability of bankruptcy using a Probit model and demonstrated that this probability lessens in accordance with return on assets and the liquidity of these, but increases with respect to leverage. [18] performed a comparison of the prediction results of the MDA and Logit methods.

The work of [19] delimits the concept of insolvency and attempts to reduce the distance between the terms of financial difficulties and bankruptcy. Thus, while “bankruptcy” covers firms in a legal situation of insolvency, “financial difficulties” usually classifies firms in accordance with solvency ratios established by a reference criterion. For example, [20] perform a multi-class classification of Chinese firms.

### Bankruptcy prediction models

In the construction of models that have tried to offer strict predictions of bankruptcy, several different studies are outstanding, with most of these centring on one particular country or industry only.

Using samples of American firms, [9] managed to attain an accuracy of 86.8% with a MDA model and 77.0% with neural networks (NN). [21] achieved 100% accuracy during the training phase and 97.5% during the testing phase with NN. [22] undertook a comparative analysis of four types of bankruptcy prediction models using ratios of financial statements, cash flows, share performance and standard deviations of that performance, achieving a classification precision of 84.9%. [10] apply a Logit model using information from the two years prior to the bankruptcy. [23] presented a model to predict the probability of bankruptcy using Logit, with which a 54% classification was attained. [24] created a model for the optimisation of the structure of firm capital with the probability of bankruptcy as the main restrictive factor. [25] investigated whether size affects the probabilities of bankruptcy by developing four discrete-time risk models (discrete-time, duration-dependent hazard model), while also using American firms as a basis for this.

Several different studies used samples of European firms. [26] carried out a process for the selection of predictive variables for bankruptcy based on decision trees (DT) and non-parametric regression. To do this, they used a sample of Slovenian firms and attained a range of accuracy between 62.8% and 95.0%. [11] managed to predict bankruptcy using financial ratios of the market and macroeconomics, applying Logit and NN to a sample of non-financial firms in the United Kingdom. In their study they detected that macroeconomic variables show some reliability problems. [27] used a series of diverse techniques (MDA, NN, DT and Logit) for Russian firms. In their models they attained a precision of 87.80%. [28] using NN, the Cox model and Logit managed to correctly predict 81.20% of bankruptcy cases, using a sample of French firms for their models. [12] developed a Logit bankruptcy prediction model based on Belgian firms, including control variables such as the size and age. The results showed that ratios of profitability and liquidity increase the accuracy of bankruptcy prediction.

For their part, [29] used case-based reasoning and NN to correctly predict between 81.5% and 83.8% of bankruptcies of Korean firms. [30] used Logit on a sample of Australian firms and correctly predicted 96% of the cases. [31] applied a Logit model on a set of data for Chinese firms, revealing that the accuracy rates for the model's predictions—both inside and outside the sample—were 97.1% and 94.1% respectively.

In the literature for bankruptcy prediction other modern classification techniques have been used, which are also capable of offering highly precise predictions. Such is the case with rough sets [32–35]; genetic algorithms [33–38]; and support vector machines [39–42]. Nevertheless, if we consider the prediction intervals, we can see that the values of the lower ranges have diminished over time. This seems to suggest that more modern methods do not always guarantee the best results. As such, there are no definitive conclusions as to which methodology is most precise for constructing models.

### Global models for insolvency prediction

As can be seen from the preceding section, the prediction of business insolvency is an area which has been extensively studied over time, although the majority of existing studies are characterised by their reference to a specific industry or a determined country, with very few global studies; that is, those using companies from different countries as a reference.

Among the studies that are global there are, in turn, different focuses, and not all of them focus exclusively on the concept of bankruptcy. The objective of a large part of these studies has been to check the effectiveness of the different prediction models. Thus, [43] developed a model to estimate the probability of financial difficulties using technical panel data. This study used the data from a sample of firms from the G-7 countries to obtain an indicator of the probability of financial difficulties that includes the specific nature of each firm.

[44], used a sample of Polish and Australian firms, proposing a bankruptcy prediction model based on the diffuse adaptation method of k-nearest neighbour. Their results confirmed that the proposed model allows the identification of the most significant financial ratios.

[45] elaborated a model with North American and Japanese firms, using, amongst other approaches, linear discriminant analysis, Logit, NN and support vector machines (SVM). This study investigated the accuracy of the bankruptcy prediction models with balanced and unbalanced samples. The results suggested that the suitable sampling method for the development of prediction models mainly depends on the number of bankruptcies in the entire training sample.

[46] developed a bankruptcy prediction model for European industrial firms based on Multilayer Perceptron (MLP). The proposed model managed to correctly predict 92.5% and 92.1% of the training and testing samples, respectively, using financial information from the two-year period prior to bankruptcy. Their conclusions suggest that European industrial firms that are less capitalised, that fail to generate sufficient resources to meet their short-term financial debt, that have low profitability and that are small in size have been most likely to suffer bankruptcy in the current financial crisis. [47], with firms from Australia, Germany and Japan, performed a comparative study of the effectiveness of different classifiers, such as MLP, SVM and DT, on the basis of well-known combination methods such as voting, bagging and boosting. The results show that DT combined with boosting is the technique that offers the greatest accuracy. [48] used a sample of Taiwanese and Chinese firms and researched the effect of the selection of the prediction models' variables using different classification techniques such as MDA, t-test, Logit, genetic algorithms and particle swarm optimisation. The results demonstrated that no single best methodological combination exists. [49] used hazard models in order to ascertain which factors determine a greater probability of financial distress for small and medium sized firms in Europe, and suggest that the location and number of shareholders are important indicators.

Other international studies have focused on studying the financial difficulties of banks in different countries. For example, [50] apply NN to a sample of US, Turkish and Spanish banks, using the data management group method. [51] and [52] apply Logit for banks in countries of the Gulf Cooperation Council and Europe, respectively.

Lastly, only two studies have had as their objective the comparison of models created for specific regions of the world [8,13]. The [13] paper focused on studying industrial firms in financial difficulties in the United States, Europe and Asia. To do this they used Logit and corresponding data one year before the situation of financial difficulty of the companies. Their conclusions indicate that regional models are superior to global models and that the differences between the regional models constructed are related to factors such as imports and exports between the countries, labour conditions and the macroeconomic setting. The study by [8] uses companies in the legal position of bankruptcy and compares the effectiveness of different prediction models between two regions, namely Latin-America (Mexico, Argentina, Brazil, Chile and Peru) and Central Europe (Poland). He builds a bankruptcy risk model with a time horizon of two years, using MDA, DT and NN, and a set of 14 financial ratios as possible predictive variables. He reached the conclusion that type I errors are greater in the Latin-American firms than the European firms, and that DT is the model which is most effective in both samples.

The above research also has other shortcomings. Firstly, global bankruptcy prediction models barely exist. The aforementioned study by [8] compares the predictive power of different techniques in two regions, but does not construct a global model capable of predicting in the different regions of the world. Similarly, the study by [13], although it tests the accuracy of a global model in different regions, does so from a wider perspective of financial distress that covers companies in financial difficulties but fails to focus on bankrupt companies. And secondly, there is a need for a precise evaluation of global models using procedures that value not only the adjustment of the model but also its complexity [53].

### Research hypotheses

From a review of the literature on bankruptcy prediction, we can see the lack of development of global models, possibly due to the difficulty in establishing representative samples for different regions of the world. From this perspective, and taking into account the pattern followed by the literature, the lines of research in this field have to be orientated towards carrying out studies seeking a greater, more global understanding of the bankruptcy problem.

Given the gap in the research, this study aims to verify whether it is possible to construct a markedly global model with a high capacity to predict bankruptcy in any region of the world. For this reason, we have formulated the following hypotheses:

Hypothesis 1 (H_1_): The predictive variables of bankruptcy in a global model are different to those in regional models.
Hypothesis 2 (H_2_): The introduction of dummy regional variables improves the predictive capacity of the global model.
Hypothesis 3 (H_3_): A global model is capable of making correct predictions in the different regions of the world.

## Model Specification and Methods

This study uses a Logit model in order to predit bankruptcy based on observable financial variables of the companies. The model specification may be represented via expression Eq (1).

$$y_{i}^{*}=x_{i}+\varepsilon_{i}$$ (1)

With:

$$y_{i}=\left\{ \begin{array}{l} 1\;if{\text{ y}}_{i}^{*}>0.5\\ 0\;if{\text{ y}}_{i}^{*}\leq 0.5\; \end{array}\right.$$

Then:

$$P[y_{i}=1]=P[x_{i}\beta +\varepsilon_{i}>0.5]=F(x_{i}\beta )$$ (2)

$$P[y_{i}=0]=1-F(x_{i}\beta )$$ (3)

As [54] mentions, models with dependent discreet variables frequently appear as index function models; that is, we interpret the result of a discrete choice as a reflection of an underlying regression. In the case of this study, the dependent variable ($y_{i}^{*}$) has a value of 1 if the company is bankrupt and has a value of 0 otherwise. We identify a company as bankrupt in accordance with the concept used by COMPUSTAT: companies that are not registered in their respective stock indexes at year end (December 31); that is, they are not quoted at market close, because they have been classified (legally declared) as bankrupt companies (code 02-Bankruptcy in Inactive Company Marker de COMPUSTAT). The parameters vector *β* reflects the impact that the independent variables vector *x*_*i*_ has on the probability of bankruptcy. The logistic distribution function is used, and the model was estimated using the backwards steps method, where the elimination of variables is based on the probability of the plausibility statistic, which, in turn, is based on estimates of maximum partial plausibility. If the probability estimate is greater than 0.5, the prediction is that it does belong to the group of bankrupt companies, otherwise it is assumed to belong to the other group considered. As such, from Eq (1):

$$P(y_{i}=1)=\frac{e^{\beta \prime x}}{1+e^{\beta \prime x}}=\frac{1}{1+e^{-(\beta \prime x)}}$$ (4)

Thus, the ratio between the two probabilities (known as the Odds ratio) would be defined as in Eq (5).

$$Odds=\frac{P(y_{i}=1)}{1-P(y_{i}=1)}=\frac{1/[1+e^{-(\beta \prime x)}]}{1/[1+e^{\left(\beta \prime x\right)}]}=\frac{1+e^{\left(\beta \prime x\right)}}{1+e^{-(\beta \prime x)}}=e^{\left(\beta \prime x\right)}$$ (5)

The estimated coefficients (*β*) represent measurements of the changes in the Odds ratio. In this regard, a positive coefficient increases the probability of the event occurring, whilst a negative value decreases the probability of its occurrence [55]. The Odds ratios may be interpreted as the number of times that the phenomenon is more likely to occur than that it is not.

By applying the logarithms in Eq (5) we obtain Eq (6), which is a linear expression of the model under consideration.

$$y_{i}{}^{*}=\text{ln}\frac{P(y_{i}=1)}{1-P(y_{i}=1)}=\text{ln}e^{\beta \prime x}=\beta \prime x$$ (6)

Taking into account that this study considers several different alternative models, the problem arises concerning which model to choose from among the series of models that have been evaluated. For this reason, we also propose criteria based on statistical information for the selection of regression models, specifically the criteria of [56–58].

The basic criterion from those based on statistical information is [56]. Generally, this criterion is expressed as in Eq (7).

AIC=2k−2Ln(L) (7)

where *k* represents the number of parameters and *L* is the maximum value of the plausibility function of the model under consideration. The basic, underlying notion behind the use of Akaike's criterion for model selection is the maximisation of the plausibility function logarithm expected for a given model.

[57] suggested that Akaike's criterion might not be asymptotically justifiable and presented an alternative information criterion using a Bayesian approach. Using this criterion, we penalise the number of parameters with *Ln (n)* instead of with *2*. Thus, the expression of Schwarz's criterion would be as shown in Eq (8).

BIC=−2Ln(L)+Ln(n)×k (8)

where *k* is the number of parameters, *L* is the maximum value of the plausibility function of the model being evaluated, and *n* the number of observations.

Finally, the criterion of [58] may be considered to be a variant of Schwarz's criterion with a minor penalisation of the magnitude of the sample size. This is specified as shown in Eq (9).

HQC=−2Ln(L)+2Ln[Ln(n)]×k (9)

where *k* is the number of parameters, *L* is the maximum value of the plausibility function of the model being evaluated, and *n* the number of observations. As in the case with the two previous criteria, we select the model that minimises the value of HQC.

## Data, Sample and Variables

This study uses a global sample of 440 non-financial, quoted companies belonging to three regions: Asia (Japan, South Korea, Singapore and Taiwan), Europe (Austria, Denmark, France, Germany, Ireland, Italy, Luxembourg, Holland, Norway, Portugal, Spain, Sweden, Switzerland and the United Kingdom) and America (Bermuda, Canada and the United States). The annual data for these companies were obtained from the COMPUSTAT database for the period 1990–2013. From the total number of companies in the sample, 220 are in a legal situation of bankruptcy and the rest of the companies (non-bankrupt) were selected randomly within those classified as actives in COMPUSTAT, following a match criteria by country, industry and year. Using this data, we formulated three different sub-samples, one for every region (S1–S14 Files).

In addition, and in order to test the predictive capability of the models, different test samples were used, and unrelated to those used in estimating the models. From a random selection, we reserved 70% of the data to build training samples, and remaining 30% to obtain testing samples.

The majority of previous studies related to bankruptcy prediction analysed data from one year prior to bankruptcy [17,34], and only a few analysed data from two or three years beforehand [8,52]. In this study, three pools of data have been built for the period 1990–2013. For bankrupt companies, the first one includes the information one year prior to bankruptcy (t-1); the second one includes data two years prior to bankruptcy (t-2); finally, the third one is based on the information three years before bankruptcy (t-3). Every pool includes information of non-bankrupt companies corresponding to years which are included information of bankrupt companies.

The number of companies used in each pool of data is shown in Table 1. Table 2 presents the distribution of the firms in the sample according to type of industry.

**Table 1:** Pooled analysis 1990/2013. Number of bankrupt and non-bankrupt firms.

Region		Total			Training data		Testing data	
		Total no. obs. for horizon	Total no. bankrupt	Total no. non- bankrupt	No. bankrupt	No. non- bankrupt	No. bankrupt	No. non- bankrupt
**ASIA**	**t-1**	96	48	48	34	34	14	14
	**t-2**	96	48	48	34	34	14	14
	**t-3**	92	46	46	32	32	14	14
	**Total horizon**	**284**	**142**	**142**	**100**	**100**	**42**	**42**
**EUROPE**	**t-1**	172	86	86	60	60	26	26
	**t-2**	172	86	86	60	60	26	26
	**t-3**	168	84	84	59	59	25	25
	**Total horizon**	**512**	**256**	**256**	**179**	**179**	**77**	**77**
**AMERICA**	**t-1**	172	86	86	60	60	26	26
	**t-2**	168	84	84	59	59	25	25
	**t-3**	160	80	80	56	56	24	24
	**Total horizon**	**500**	**250**	**250**	**175**	**175**	**75**	**75**
**GLOBAL**	**t-1**	440	220	220	154	154	66	66
	**t-2**	436	218	218	152	152	66	66
	**t-3**	420	210	210	147	147	63	63
	**Total horizon**	**1296**	**648**	**648**	**453**	**453**	**195**	**195**

**Table 2:** Industries distribution of the sample.

GICS*	10	15	20	25	30	35	45	50	55	Total
Asia (t-1)	-	6	32	36	6	-	16	-	-	96
Asia (t-2)	-	6	32	36	6	-	16	-	-	96
Asia (t-3)	-	6	32	34	4	-	16	-	-	92
Europe (t-1)	4	6	40	46	12	-	52	8	4	172
Europe (t-2)	4	6	42	44	12	-	52	8	8	172
Europe (t-3)	4	6	40	40	14	-	48	10	6	168
America (t-1)	14	14	22	64	8	20	22	2	6	172
America (t-2)	14	14	22	62	8	20	20	2	6	168
America (t-3)	12	14	22	56	8	20	20	2	6	160
Global (t-1)	18	26	94	146	26	20	90	10	10	440
Global (t-2)	18	26	96	142	26	20	88	10	10	436
Global (t-3)	16	26	94	130	26	20	84	12	12	420

*GICS: 10-Energy-, 15-Materials-, 20-Industrials-, 25-Consumer discretionary-, 30-Consumer staples-, 35-Health care-, 45-Information technology-, 50-Telecommunication services-, 55-Utilities-.

The majority of bankruptcy prediction studies have used financial variables as independent variables. In this study, we utilised the 10 most used financial variables in the existing, prior literature, which cover aspects of profitability (V1, V4, V5, V10), debt (V8), liquidity (V2, V3, V7, V9) and efficiency (V6) [59]. In addition, dummy variables are included to identify the industry to which the company belongs (in accordance with the classification by COMPUSTAT using the Global Industry Classification Standard—GICS), and to show the region to which they belong. Table 3 shows the econometric variables used in the study.

**Table 3:** Econometric variables definition.

Earnings/Total assets	V1
Current assets/Current liabilities	V2
Working capital/Total assets	V3
Retained earnings/Total assets	V4
EBIT/Total assets	V5
Sales/Total assets	V6
(Current assets + Cash flow)/Current liabilities	V7
Total debt/Total assets	V8
Current assets/Total assets	V9
Earnings/Net worth	V10
GICS	V11
1: Asia, 2: Europe, 3: America	Region

Financial data expressed in nominal value.

## Results

### Descriptive statistics

The principle descriptive statistics (average and standard deviation) for the variables for the regions under analysis are shown in Tables 4, 5 and 6. In general, the average values of the variables in non-bankrupt firms are higher than those for bankrupt firms, except in the case of debt. This behaviour occurs in all the regions analysed. As such, non-bankrupt firms are more profitable and more efficient, but have less debt than bankrupt firms.

**Table 4:** Descriptive statistics. Asia.

		V1	V2	V3	V4	V5	V6	V7	V8	V9	V10
(t-1)	Non-bankrupt firms	0.032 (0.027)	1.640 (0.689)	0.194 (0.150)	0.229 (0.196)	0.055 (0.039)	1.123 (0.555)	1.145 (0.563)	0.243 (0.165)	0.572 (0.177)	0.073 (0.075)
	Bankrupt firms	-0.210 (0.391)	0.983 (0.587)	-0.138 (0.401)	-0.268 (0.484)	-0.043 (0.120)	1.039 (0.514)	0.665 (0.534)	0.549 (0.341)	0.562 (0.212)	-0.498 (3.106)
(t-2)	Non-bankrupt firms	0.032 (0.027)	1.640 (0.689)	0.194 (0.150)	0.229 (0.196)	0.055 (0.039)	1.123 (0.555)	1.145 (0.563)	0.243 (0.165)	0.572 (0.177)	0.073 (0.075)
	Bankrupt firms	-0.045 (0.158)	1.107 (0.468)	0.018 (0.236)	-0.045 (0.257)	-0.025 (0.129)	0.978 (0.442)	0.756 (0.476)	0.430 (0.224)	0.582 (0.217)	-0.204 (0.628)
(t-3)	Non-bankrupt firms	0.033 (0.026)	1.627 (0.701)	0.185 (0.146)	0.238 (0.186)	0.057 (0.039)	1.093 (0.547)	1.135 (0.572)	0.246 (0.164)	0.560 (0.170)	0.076 (0.075)
	Bankrupt firms	-0.057 (0.115)	1.112 (0.442)	0.024 (0.228)	-0.021 (0.180)	-0.007 (0.063)	0.996 (0.443)	0.744 (0.417)	0.417 (0.212)	0.580 (0.203)	-0.139 (0.806)

Standard deviation in brackets.

**Table 5:** Descriptive statistics. Europe.

		V1	V2	V3	V4	V5	V6	V7	V8	V9	V10
(t-1)	Non-bankrupt firms	0.039 (0.098)	1.659 (0.869)	0.157 (0.195)	0.082 (0.386)	0.081 (0.078)	1.115 (0.578)	1.169 (0.724)	0.187 (0.149)	0.479 (0.210)	0.074 0.288
	Bankrupt firms	-0.190 (0.277)	1.174 (0.748)	-0.007 (0.318)	-0.210 (0.468)	-0.132 (0.227)	1.062 (0.646)	0.734 (0.454)	0.363 (0.246)	0.537 (0.210)	-0.324 (2.372)
(t-2)	Non-bankrupt firms	0.036 (0.140)	1.953 (1.902)	0.177 (0.237)	0.069 (0.691)	0.079 (0.100)	1.140 (0.577)	1.358 (1.231)	0.187 (0.169)	0.506 (0.215)	0.101 (0.201)
	Bankrupt firms	-0.122 (0.344)	1.556 (0.944)	0.141 (0.257)	-0.313 (1.490)	-0.091 (0.294)	0.987 (0.634)	1.044 (0.835)	0.283 (0.193)	0.587 (0.216)	-0.294 (0.740)
(t-3)	Non-bankrupt firms	0.034 (0.141)	1.955 (1.915)	0.175 (0.241)	0.019 (0.705)	0.075 (0.100)	1.121 (0.571)	1.341 1.230	0.200 (0.175)	0.510 (0.214)	0.098 (0.203)
	Bankrupt firms	-0.051 (0.210)	1.850 (1.393)	0.188 (0.250)	-0.055 (0.363)	-0.016 (0.193)	1.172 (0.743)	1.240 (1.077)	0.274 (0.203)	0.599 (0.204)	-0.040 (0.467)

Standard deviation in brackets.

**Table 6:** Descriptive statistics. America.

		V1	V2	V3	V4	V5	V6	V7	V8	V9	V10
(t-1)	Non-bankrupt firms	0.037 (-0.254)	2.257 (1.609)	0.225 (0.099)	0.605 (-1.696)	0.080 (-27.605)	1.079 (1.107)	1.329 (1.030)	0.261 (0.504)	0.434 (0.422)	0.150 (-316.325)
	Bankrupt firms	0.070 (0.439)	1.142 (1.874)	0.187 (0.216)	0.557 (9.012)	0.096 (0.327)	0.679 (0.820)	0.828 1.738	0.188 (0.485)	0.217 (0.240)	0.395 (8.818)
(t-2)	Non-bankrupt firms	0.039 (-0.146)	2.268 (1.883)	0.224 (0.108)	0.077 (-6.038)	0.081 (-0.074)	1.076 (1.139)	1.334 (1.229)	0.266 (0.403)	0.431 (0.418)	0.155 (-2.915)
	Bankrupt firms	0.072 (0.352)	1.153 (2.273)	0.189 (0.252)	0.549 (45.021)	0.097 (0.243)	0.683 (0.840)	0.836 (2.178)	0.187 (0.396)	0.218 (0.230)	0.400 (28.904)
(t-3)	Non-bankrupt firms	0.038 (-0.123)	2.369 (1.565)	0.234 (0.099)	0.061 (-1.696)	0.082 (-0.074)	1.048 (1.093)	1.436 (1.011)	0.269 (0.404)	0.439 (0.399)	0.157 (-9.966)
	Bankrupt firms	0.073 (0.425)	1.328 (0.818)	0.197 (0.217)	0.558 (9.012)	0.099 (0.328)	0.652 (0.754)	0.983 (0.704)	0.188 (0.289)	0.221 (0.226)	0.407 (88.181)

Standard deviation in brackets.

On the other hand, the analysis of the global sample presents us with characteristics similar to those detected for the regional samples (Table 7). Non-bankrupt firms continue to present average values higher than those of bankrupt firms. However, in this case there are two exceptions: efficiency (V6) and liquidity (V9).

**Table 7:** Descriptive statistics. Global sample.

		V1	V2	V3	V4	V5	V6	V7	V8	V9	V10
(t-1)	Non-bankrupt firms	0.037 (-0.261)	1.895 (1.219)	0.202 (0.120)	0.083 (-0.662)	0.070 (-15.536)	1.092 (1.093)	1.246 (0.777)	0.229 (0.477)	0.496 (0.506)	0.101 (-3.726)
	Bankrupt firms	0.073 (0.410)	1.002 (1.231)	0.204 (0.276)	0.569 (54.189)	0.088 (0.234)	0.604 (0.711)	0.767 (1.078)	0.178 (0.384)	0.211 (0.232)	0.294 (5.352)
(t-2)	Non-bankrupt firms	0.033 (-0.133)	2.024 (1.586)	0.200 (0.101)	0.106 (-2.282)	0.072 (-0.071)	1.095 (1.013)	1.296 (1.036)	0.230 (0.371)	0.491 (0.525)	0.105 (-1.204)
	Bankrupt firms	0.105 (0.334)	1.487 (1.589)	0.208 (0.284)	0.556 (26.220)	0.091 (0.240)	0.613 (0.693)	0.961 1.418	0.179 (0.297)	0.215 (0.233)	0.304 (16.945)
(t-3)	Non-bankrupt firms	0.033 (-0.09)	2.030 (1.656)	0.198 (0.120)	0.083 (-0.662)	0.071 (-0.039)	1.075 (1.059)	1.327 (1.124)	0.235 (0.358)	0.492 (0.521)	0.106 (-3.726)
	Bankrupt firms	0.107 (0.299)	1.514 (1.795)	0.211 (0.277)	0.570 (5.419)	0.092 (0.234)	0.596 (0.695)	1.020 (1.449)	0.180 (0.261)	0.213 (0.237)	0.309 (53.526)

Standard deviation in brackets.

### Model Development

In order to verify the proposed research hypotheses different regression models have been constructed. Thus, and to check whether the bankruptcy prediction variables in a global model are different to those of the regional models (H_1_), four regressions were constructed, three of these with the regional samples and a fourth with the global sample. The specifications of these models appear in Table 8.

**Table 8:** Regional and Global Models (hypothesis 1).

Specification Models			Summary				Classification Accuracy (%)	
			Omnibus Test	Hosmer-L. Test	ROC Curve	R^2^ Nagelk.	Training Sample	Testing Sample
**ASIA**	t-1	*Y*_(AS) t-1_ = -2.323–30.147V1*** - 13.413V4*** + 8.385V8**	0.000***	1.000***	0.958	0.814	91.07	89.29
	t-2	*Y*_(AS) t-2_ = 0.140–30.181V1** -5.177V4** -15.086V5* + 3.934V8**	0.000***	0.947***	0.895	0.624	81.56	89.29
	t-3	*Y*_(AS) t-3_ = 2.996–43.695V1*** -1.784V2**	0.000***	0.925***	0.929	0.696	83.76	82.14
**EUROPE**	t-1	*Y*_(E) t-1_ = 1.899 + 10.532V1** - 1.812V2*** - 25.680V5***+8.059V8*** + 5.626V9***	0.000***	0.761***	0.904	0.738	85.81	92.57
	t-2	*Y*_(E) t-2_ = 1.046–1.959V2** + 9.923V3*** + 1.722V4*** -20.098V5*** + 4.360V8**	0.000***	0.310***	0.846	0.536	78.81	87.53
	t-3	*Y*_(E) t-3_ = -1.465 + 1.852V3* + 2.166V4*** - 16.299V5*** + 0.803V6** + 3.468V8**	0.000***	0.310***	0.840	0.340	72.70	81.67
**AMERICA**	t-1	*Y*_(A) t-1_ = -1.389–10.636V3*** - 19.907V5*** + 1.175V6**	0.000***	0.499***	0.938	0.722	87.39	87.23
	t-2	*Y*_(A) t-2_ = -1.507–3.466V3** - 13.383V5*** + 0.985V6*** + 3.767V8***	0.000***	0.056**	0.843	0.510	82.14	84.88
	t-3	*Y*_(A) t-3_ = -1.425–3.516V3** - 1.717V5*** + 1.226V6*** + 3.251V8***	0.000***	0.955***	0.801	0.338	80.37	80.45
**GLOBAL**	t-1	*Y*_(G) t-1_ = -2.353–4.762V3*** - 17.929V5*** + 4.246V8*** + 2.721V9**	0.000***	0.601***	0.906	0.653	83.72	84.86
	t-2	*Y*_(G) t-2_ = -1.215–0.404V2*** - 12.151V5*** + 3.440V8*** + 2.267V9**	0.000***	0.422***	0.885	0.417	79.19	79.50
	t-3	*Y*_(G) t-3_ = -1.240–10.808V5*** + 0.634V6*** + 2.909V8***	0.000***	0.567***	0.817	0.293	74.91	74.89

**Sig. at 0.05

***Sig. at 0.01

The bankruptcy prediction models for Asia mainly consist of three variables: Earnings/Total assets (V1), Retained earnings/Total assets (V4) and Total debt/Total assets (V8). As such, they select as the best bankruptcy predictors the variables that refer to profitability and debt.

In the case of the models for Europe, the significant variables turned out to be Earnings/Total assets (V1), Current assets/Current liabilities (V2), EBIT/Total assets (V5), Total debt/Total assets (V8) and Current assets/Total assets (V9). Hence, the aspects of profitability, debt and liquidity turned out to be more important when predicting bankruptcy in European firms.

For the models constructed with American companies, the significant variables refer to liquidity, profitability, efficiency and debt. More specifically, the following variables were chosen: Working capital/Total assets (V3), EBIT/Total assets (V5), Sales/Total assets (V6), and Total debt/ Total assets (V8).

The global models are formed by the variables Current assets/Current liabilities (V2), Working capital/Total assets (V3), EBIT/Total assets (V5), Sales/Total assets (V6), Total debt/ Total assets (V8), and Current assets/Total assets (V9). These global models also cover aspects of liquidity, profitability, efficiency and debt, but the set of variables for t-1, t-2, and t-3 do not coincide with any of the regional models.

All of the models constructed attain a high percentage of accuracy (greater than 80%). Moreover, both the Omnibus test and the Hosmer and Lemeshow test, and the R^2^ indicate that the goodness-of-fit of the estimate of these is acceptable. The area beneath the ROC curve (in all cases very close to 1) confirms that the four models correctly classify bankrupt firms and non-bankrupt firms.

A comparison between the results of the global model and the three regional models constructed allowed us to detect that there are differences between these. As a consequence, hypothesis H_1_ is accepted, as the regional models are different to the global model.

In order to check hypothesis H_2_, which proposes that the introduction of a regional perturbation (which covers those unobservable impacts brought about by factors that are particular to each region) improves the predictive capacity of the global model, three new models were constructed using the global firms sample. In this case, a dummy variable (Region) was included among the independent variables, which refers to each one of the regions considered in this study (1 for Asia, 2 for Europe and 3 for America). The results of the global models with these dummy variables can be seen in Table 9. In all of the models, the following were selected as significant variables: Working capital/Total assets (V3), EBIT/Total assets (V5), Current assets/ Total assets (V9), and Region. As such, in addition to the geographical location, the aspects of liquidity and profitability are also represented.

**Table 9:** Global Model with Regional Dummy (hypothesis 2).

Coefficients and Variables		Summary				Classification Accuracy (%)	
		Omnibus Test	Hosmer- L. Test	ROC Curve	R^2^ Nagelk.	Training Sample	Testing Sample
**t-1**	*Y*_(Gd)t-1_ = -2.695 + 6.635V3*** - 18.292V5*** + 4.759V9*** + 0.713Region***	0.000***	0.688***	0.931	0.640	84.51	90.11
**t-2**	*Y*_(Gd)t-2_ = -2.343–0.767V2*** - 11.426V5*** + 3.624V8*** + 3.385V9*** + 0.502Region**	0.000***	0.220***	0.890	0.441	77.37	84.35
**t-3**	*Y*_(Gd)t-3_ = -2.867–2.728V3*** - 9.992V5*** + 2.833V8*** + 3.633V9*** + 0.446Region**	0.000***	0.414***	0.817	0.314	72.63	78.85

**Sig. at 0.05

***Sig. at 0.01

In Table 9 we can also see that the adjustment and the accuracy of these global models with regional dummy are acceptable in all cases. In comparison to the results obtained before with global models without regional dummy (Table 8), accuracy is now greater, with percentages of 90.11%; 84.35% and 78.85% for the samples t-1, t-2 and t-3, respectively.

Lastly, and in order to determine whether the global model with regional dummy is better at predicting bankruptcy than the global model without regional dummy, we used the criteria of [56–58]. The results are shown in Table 10. For the three criteria used, the best model is the one using the regional dummy. Consequently, hypothesis H_2_ is also accepted.

**Table 10:** Selection Tests for Global Models (hypothesis 2).

		Global Model	Global Model with Regional Dummy
t-1	AIC	227.072	103.423
	BIC	231.844	123.283
	HQC	223.106	110.347
t-2	AIC	292.723	118.933
	BIC	297.485	145.138
	HQC	288.757	128.101
t-3	AIC	313.614	123.377
	BIC	317.153	150.423
	HQC	310.628	133.508

AIC: Akaike, BIC: Bayesian, HQC: Hannan-Quinn

With regard to the hypothesis H_3_, where the global model is applied using the samples of each region, the accuracy of the global model was checked with regional dummy in the three regional test samples. The results obtained are shown in Table 11. It can be seen that in all cases this global model offers acceptable predictions, and that the accuracy percentages obtained are considerably better than those for the regional models (Table 8). In addition, according to the Akaike, Bayesian, and Hannan-Quinn criteria the global model is better than the regional models (Table 12). As such, hypothesis H_3_ is also accepted.

**Table 11:** Results of Global Model using data from Regions (hypothesis 3).

	t-1			t-2			t-3		
	Asia	Europe	America	Asia	Europe	America	Asia	Europe	America
**Summary**									
Omnibus Test	0.000	0.000	0.000	0.000	0.000	0.000	0.000	0.000	0.005
Hosmer- L. Test	0.964	0.239	0.646	0.961	0.753	0.180	0.600	0.090	0.276
R^2^ Nagelkerke	0.915	0.679	0.657	0.834	0.747	0.475	0.798	0.572	0.428
ROC Curve	0.962	0.942	0.941	0.929	0.904	0.863	0.936	0.840	0.813
**Classification Accuracy (%)**									
Total	92.92	94.13	89.72	90.02	91.79	86.52	92.63	87.22	82.14

**Table 12:** Models Selection Tests (hypothesis 3).

		Regional Models			Global Model using data from Regions		
		Asia	Europe	America	Asia	Europe	America
t-1	AIC	16.377	38.407	39.765	12.386	38.274	39.722
	BIC	27.567	60.152	52.864	25.315	51.321	52.651
	HQC	20.851	45.563	44.746	16.860	43.363	43.538
t-2	AIC	34.661	45.718	56.082	20.077	42.398	55.723
	BIC	49.581	67.463	73.321	34.997	59.794	72.962
	HQC	40.626	54.201	64.767	24.658	49.184	62.460
t-3	AIC	25.964	62.500	57.711	20.846	55.734	55.734
	BIC	33.424	83.799	74.950	32.036	68.663	68.541
	HQC	28.946	70.671	64.396	26.749	53.416	61.838

AIC: Akaike, BIC: Bayesian, HQC: Hannan-Quinn

The results obtained assume the existence of a global model for bankruptcy prediction. These results may be explained by evidence from previous research which holds that bankruptcy risk depends more on global effects than on the country effect [1]. Nevertheless, our results are different to those obtained by previous research on global models for the prediction of financial distress. In this case, [13] rejected the hypothesis of a global model in favour of individual models for each region. Even so, the results obtained here may complement those of [13], helping us to understand that even if regional models are better at detecting possible situations of financial distress, we can use a global model to predict bankruptcy situations.

In this research we have also verified that international bankruptcy prediction is not influenced by the industry to which the company belongs, as the GICS variable has not proven to be significant in any of the global models constructed. Nevertheless, [1] detected that changes to insolvency risk at a global level also depend on the industry in which the firm operates. These differences in results are possibly due to the use of different concepts of insolvency, with a much wider concept used in the [1] study and a much more restrictive one in our research.

## Conclusions and Implications

The recent world financial crisis has increased the number of bankruptcies in numerous countries and has brought about a new area of research, given the need to create models to predict this phenomenon not only at a country level, but also at a global level. This need seems to be a consequence of the globalisation process, which has resulted in the greater homogenisation of firms' financial behaviour.

In the field of predicting business insolvency, models that have attempted to offer strict bankruptcy predictions have focused on companies in individual countries or industries, with an absence of studies that consider the problem of bankruptcy from a global perspective. This study uses samples of bankrupt firms from 3 regions over a time period of up to 3 years to try and determine whether a common pattern exists that can explain the bankruptcy process in any part of the world.

The results obtained have allowed us to confirm the superiority of a global model of bankruptcy prediction in comparison to regional models. Although regional differences do exist for indicators, in relation to bankruptcy the differences are not so great, as the regional models coincide in many aspects. Nonetheless, this convergence of bankruptcy indicators finds its maximum expression in the global model, which gathers information (experience) from multiple regions and is then capable of projecting this with a great deal of accuracy.

Our evidence helps to explain that the globalisation process extends from the financial characteristics of firms to the factors that cause bankruptcy. These conclusions may be important when minimising the cost of constructing bankruptcy prediction models, given the existence of explanatory financial variables which are common to the most important regions in the world. In addition, and due to the power of generalisation demonstrated by the global model, we emphasise the need for multinational firms to manage their own bankruptcy prediction models, applying them to clients, suppliers and the firms in which they have holdings. Lastly, the existence of a global model for bankruptcy prediction can also meet the requirements of International Standards on Auditing with respect to the going concern principle, which proposes the use of feasibility models for firms in order to support auditors' opinions in the context of risk assessment.

Like all research, this study has some limitations, mainly the availability of firms data in emerging countries. Given that it is research undertaken from a global perspective, it requires a much greater scope of information in comparison to other studies performed in this field. In addition, future research could set an approach to investigate which macro conditions affect the behavior of the financial variables that have proved as good predictors bankruptcy in this paper. Finally, to increase the generalizability of the results, data from other firms (i.e., small and medium-sized enterprises) should be included.

## Supporting Information

S1 File Asiat1. Variables of bankrupt/non-bankrupt Asian firms in t-1.
(XLSX)

S2 File Asiat2. Variables of bankrupt/non-bankrupt Asian firms in t-2.
(XLSX)

S3 File Asiat3. Variables of bankrupt/non-bankrupt Asian firms in t-3.
(XLSX)

S4 File Data Sheet. Variables description according to S&P’s COMPUSTAT information.
(PDF)

S5 File Europet1. Variables of bankrupt/non-bankrupt European firms in t-1.
(XLSX)

S6 File Europet2. Variables of bankrupt/non-bankrupt European firms in t-2.
(XLSX)

S7 File Europet3. Variables of bankrupt/non-bankrupt European firms in t-3.
(XLSX)

S8 File Globalt1. Variables of bankrupt/non-bankrupt Global firms in t-1.
(XLSX)

S9 File Globalt2. Variables of bankrupt/non-bankrupt Global firms in t-2.
(XLSX)

S10 File Globalt3. Variables of bankrupt/non-bankrupt Global firms in t-3.
(XLSX)

S11 File North Americat1. Variables of bankrupt/non-bankrupt North American firms in t-1.
(XLSX)

S12 File North Americat2. Variables of bankrupt/non-bankrupt North American firms in t-2.
(XLSX)

S13 File North Americat3. Variables of bankrupt/non-bankrupt North American firms in t-3.
(XLSX)

S14 File Read me. Source Information.
(TXT)
