# Correction: A Global Model for Bankruptcy Prediction

**DOI:** 10.1371/journal.pone.0208476

**Published:** 2018-11-28

**Authors:** David Alaminos, Agustín del Castillo, Manuel Ángel Fernández

There are errors in the author affiliations. The affiliations should appear as shown here: David Alaminos^1,2^, Agustín del Castillo^1^, Manuel Ángel Fernández^1^

**1** Department of Finance and Accounting, Universidad de Málaga, Málaga, Spain, **2** PhD Program in Economics and Business, Universidad de Málaga, Málaga, Spain
