# Supplementary material for: A Global Model for Bankruptcy Prediction
Source: PLoS One. 2016 Nov 23;11(11):e0166693. doi: 10.1371/journal.pone.0166693 (PMC5120822; doi:10.1371/journal.pone.0166693)
Supplement: S4 File — Variables description according to S&P’s COMPUSTAT information. (PDF) [file pone.0166693.s004.pdf]

A Global Model for Bankruptcy Prediction

Data Sheet

Code of Variable	Variable	S&P´s Compustat Information (Mnemonic*/Item Number (or Concept Calculation)**)
V1	Earnings/Total Assets	Income Before Extraordinary Items (IB/G378)
		Assets-Total (AT/G107)
V2	Current Assets/Current Liabilities	Current Assets-Total (ACT/G638)
		Current Liabilities-Total (LCT/G650)
V3	Working Capital/Total Assets	Working Capital (WCAP/ACT-LCT)
		Assets-Total (AT/G107)
V4	Retained Earnings/Total Assets	Retained Earnings (RE/G182)
		Assets-Total (AT/G107)
V5	EBIT/Total Assets	Earnings Before Interest and Taxes (EBIT/PI+XINT)
		Assets-Total (AT/G107)
V6	Sales/Total Assets	Sales-Turnover(Net) (SALE/G608)
		Assets-Total (AT/G107)
V7	(Current Assets+Cash Flow)/Current Liabilities	Current Assets-Total (ACT/G638) + Cash Flow (CFL/IB+DP)
		Current Liabilities-Total (LCT/G650)
V8	Total Debt/Total Assets	Debt-Total (DT/DlTT+DLC)
		Assets-Total (AT/G107)
V9	Current Assets/Total Assets	Current Assets-Total (ACT/G638)
		Assets-Total (AT/G107)
V10	Earnings/Net Worth	Income Before Extraordinary Items (IB/G378)
		Stockholders´Equity-Total (SEQ/G193)
V11	GICS	GICS Group-level data (GGROUP/ @LEFT(SPGICX,4)
-	Dependent Variable: Bankruptcy	1 if Inactive Company Marker-02-Bankruptcy (INCO/G715) and 0 otherwise
	**Total**	**998**

Source: S&P´s COMPUSTAT

**Mnemonic: Identification abreviation of information within S&P´s COMPUSTAT*

***Item number: Identification code of information within S&P´s COMPUSTAT*
